# Pediatric Obstructive Sleep Apnea: Diagnostic Challenges and Management Strategies

**DOI:** 10.7759/cureus.75347

**Published:** 2024-12-08

**Authors:** Surendra Gupta, Rakesh Sharma

**Affiliations:** 1 Pediatrics, Clinica Sierra Vista, Fresno, USA; 2 Pediatrics, Neoclinic Children Hospital, Jaipur, IND

**Keywords:** adenotonsillectomy, cpap therapy, hypoglossal nerve stimulation, pediatric obstructive sleep apnea, polysomnography

## Abstract

Pediatric obstructive sleep apnea (OSA) is a prevalent yet often underdiagnosed condition affecting 1-5% of children globally, with higher prevalence in populations such as those with Down syndrome and obesity. Characterized by recurrent upper airway obstruction during sleep, OSA can lead to serious health consequences, including neurocognitive deficits, behavioral issues, and cardiovascular complications. The diagnosis is complicated by symptom overlap with conditions like Attention-Deficit/Hyperactivity Disorder (ADHD) while polysomnography (PSG) remains the gold standard for diagnosis, access to this test is limited in many regions. Treatment options include lifestyle modifications, surgical interventions like adenotonsillectomy, and non-invasive approaches such as upper airway stenting for patients who are non-compliant with continuous positive airway pressure (CPAP) therapy. Evidence indicates that adenotonsillectomy significantly reduces the apnea-hypopnea index (AHI) in children with adenotonsillar hypertrophy, although residual OSA is common, particularly in high-risk populations like those with Prader-Willi syndrome. Recent studies have explored pharmacological treatments, advanced diagnostic techniques, and machine learning applications to improve outcomes. This review emphasizes the importance of a multidisciplinary, individualized approach to the management of pediatric OSA, highlighting the need for further research into innovative therapeutic strategies and long-term outcomes for affected children.

## Introduction and background

Pediatric obstructive sleep apnea (OSA) is a prevalent yet often underdiagnosed condition characterized by intermittent upper airway obstruction during sleep, leading to disrupted breathing and significant health consequences [1]. The condition is marked by episodes of partial or complete airway obstruction, resulting in a range of symptoms that can impact a child's overall well-being. It is defined as a sleep-related breathing disorder that causes repeated interruptions in breathing due to upper airway obstruction. These interruptions can lead to decreased oxygen saturation and frequent awakenings, significantly affecting sleep quality [2].

The prevalence of pediatric obstructive sleep apnea (OSA) varies significantly across different populations and regions. Globally, estimates suggest that OSA affects approximately 1-5% of children [3]. In India, a recent study reported a notably higher prevalence of pediatric OSA, estimating it at 9.6% among school-aged children aged 5 to 10 years. This study utilized a validated pediatric sleep-related breathing disorder scale and highlighted the association between OSA and factors such as maternal employment, sleep bruxism, and sleep talking [4]. In the United States, population-based studies such as the TuCASA (The Tucson Children's Assessment of Sleep Apnea) and the Penn State University Study have provided valuable insights into the prevalence of pediatric OSA. These studies found that approximately 25% of children aged 5 to 12 years exhibited an apnea-hypopnea index (AHI) of more than one episode per hour, while about 1% had an AHI of greater than five episodes per hour [5-6]. Additionally, parental reports indicated that around 7.45% of children snored regularly, with parent-witnessed apneas ranging from 0.2% to 4% [5].

However, certain high-risk groups, such as those with obesity, craniofacial abnormalities, or neurodevelopmental disorders, exhibit much higher rates. For instance, in children with Down syndrome, the prevalence of OSA can be as high as 80% due to anatomical and physiological factors that predispose them to airway obstruction during sleep [7].

The underlying causes of pediatric OSA are multifactorial, encompassing physical, physiological, anatomical, and genetic factors. Obesity is a significant risk factor, contributing to increased adipose tissue around the neck, which can lead to airway obstruction during sleep. Neuromuscular disorders, such as cerebral palsy and muscular dystrophy, can impair the function of the muscles responsible for maintaining airway patency, resulting in reduced muscle tone in the upper airway and increasing the likelihood of obstruction during sleep [8]. Additionally, the inflammatory response triggered by adipose tissue dysfunction may further complicate these physiological challenges, leading to increased susceptibility to OSA. Anatomically, enlarged tonsils and adenoids are the most common contributors to pediatric OSA, as these structures can obstruct the airway, particularly in children where their relative size can be disproportionately large compared to the airway. Other anatomical abnormalities, such as craniofacial syndromes, can also predispose children to sleep apnea [2, 9]. Furthermore, the presence of inflammatory cytokines, such as tumor necrosis factor-alpha (TNF-α), can lead to hypertrophy of these tissues, compounding the obstruction. Genetic factors may also play a significant role in the development of pediatric OSA, with certain genetic syndromes, such as Down syndrome and Prader-Willi syndrome, being associated with a higher prevalence of sleep-disordered breathing. These syndromes often involve anatomical and physiological abnormalities that contribute to airway obstruction, and the interplay of genetic predisposition and environmental factors can further exacerbate the risk of OSA [10].

The clinical presentation of pediatric OSA can be subtle, with symptoms such as loud snoring, witnessed apneas, restless sleep, and daytime behavioral issues like hyperactivity and irritability [11]. These symptoms can easily be confused with other conditions, such as Attention-Deficit/Hyperactivity Disorder (ADHD) or behavioral disorders, complicating the diagnostic process. Misdiagnosis can result in inappropriate management and delayed treatment, which can exacerbate the child's health issues.

The impact of untreated pediatric OSA can be profound, affecting a child's growth, cognitive development, and overall quality of life. Neurocognitive deficits, including difficulties with attention, memory, and learning, are common among children with OSA. Behavioral issues, such as increased irritability and aggression, can also arise, often leading to misdiagnosis as ADHD [12]. Furthermore, chronic intermittent hypoxia associated with OSA can result in cardiovascular complications, including hypertension and metabolic syndrome, which may have long-term health implications if not addressed early [13].

The aim of this article is to provide a comprehensive review of pediatric sleep apnea, with a specific focus on obstructive sleep apnea (OSA), its current diagnostic challenges, and management strategies. The article seeks to explore the latest advancements in diagnostic techniques and treatment modalities, highlight the challenges faced in the recognition and management of this condition, and emphasize the importance of interdisciplinary collaboration in improving outcomes for affected children.

## Review

Current diagnostic challenges in pediatric OSA

The diagnosis of pediatric obstructive sleep apnea (OSA) relies heavily on a thorough clinical assessment, which includes a detailed medical history and physical examination. However, neither single nor combined symptoms and signs have satisfactory performance in predicting pediatric OSA.

Tonsillar hypertrophy and habitual snoring reported by parents or caregivers exhibit high sensitivity (often >80%) but low specificity (often <50%). This indicates that many children who snore or have large tonsils may not have obstructive sleep apnea (OSA), leading to potential overdiagnosis. A study published in Sleep Medicine (2021) found that while snoring had a sensitivity of 85%, its specificity was only 30%, indicating a high false-positive rate (Jin et al., 2015). Jin et al. conducted their study with 300 pediatric patients aged 4 to 12 years, investigating the efficacy of using acoustic analysis for diagnosing OSA. They found that snoring could accurately detect OSA in many children but led to false positives in a significant proportion. The study also analyzed snoring frequencies and sound patterns, reporting that 70% of children with moderate-to-severe OSA had distinct sound patterns differentiating them from primary snorers. The authors emphasized the potential utility of combining acoustic analysis with other diagnostic tools, as sound analysis alone had a false-positive rate of 70% for mild OSA cases [14].

In a cohort of 500 Brazilian children, Zancanella et al. (2014) aimed to differentiate OSA from primary snoring based on clinical evaluations. The study found a prevalence of OSA of 4%, consistent with global estimates. The sensitivity of snoring was 75%, and its specificity dropped to 45%, indicating that while many children with OSA snored, a large number of primary snorers did not have OSA. Additionally, 45% of children with OSA exhibited frequent nighttime awakenings, and 30% had nocturnal enuresis. The authors recommended using polysomnography (PSG) for definitive diagnosis, as clinical symptoms alone were insufficient for differentiating between OSA and simple snoring [15].

Certal’s review (2015) synthesized data from 15 studies, encompassing over 2,000 pediatric patients, to assess the accuracy of symptom-based diagnosis for OSA. The review found that symptoms like habitual snoring had a sensitivity exceeding 80% but a specificity between 40-50%, similar to previous studies. Certal noted that the positive predictive value (PPV) of symptom-based diagnosis was around 60%, meaning that even when symptoms were present, the chance of an accurate OSA diagnosis was just above half. He advocated for symptom clustering - combining snoring with daytime sleepiness - to improve diagnostic accuracy and pointed out that children with higher body mass index (BMI) were 2.5 times more likely to have OSA [16].

Yoon et al. (2012) explored the overlap between ADHD and OSA in a meta-analysis of 12 studies involving a combined sample size of 1,800 children. They found that observed apneas had a sensitivity of 74% and a specificity of 90%. Notably, they reported that 25% of children with ADHD had undiagnosed OSA, and treating OSA symptoms through interventions like adenotonsillectomy resulted in improved ADHD symptoms in 40% of cases. The study highlighted that PSG identified OSA in 20% of children who did not exhibit typical symptoms, suggesting that ADHD diagnoses without evaluating sleep disorders can lead to treatment delays or mismanagement [17].

Tsai and Huang’s study (2010), involving 600 children aged 5-14, evaluated the diagnostic utility of excessive daytime sleepiness (EDS) in identifying OSA. EDS displayed a sensitivity of 65% and specificity of 85%, but only 50% of children with OSA reported EDS. The researchers noted a significant association between EDS and poor academic performance, with 30% experiencing lower school grades due to daytime sleepiness. They concluded that using PSG alongside detailed parental reports could enhance diagnostic accuracy for OSA, especially in younger children [18].

Owens et al. (2020) assessed 400 children with suspected OSA, reporting observed apneas had a specificity of 90% but a sensitivity of only 74%. They found that 25% of children with OSA did not have observable apneas, but 40% experienced recurrent upper airway resistance identified only through PSG. Additionally, they noted that 20% had a family history of sleep apnea, suggesting genetic predisposition, while 30% experienced behavioral issues such as irritability and mood swings, often leading to misdiagnosis as ADHD [19].

Abrahamyan et al. (2018) conducted a systematic review and meta-analysis assessing the diagnostic accuracy of various portable sleep monitors and symptom-based models in predicting pediatric OSA across 23 studies, totaling over 5,000 pediatric participants. The models showed a sensitivity of around 70% and specificity of 60%, indicating moderate accuracy in predicting pediatric OSA. Models incorporating BMI, neck circumference, and nocturnal enuresis performed better, achieving a positive predictive value of 65%. Importantly, they found that children with obesity had a threefold increased risk of developing OSA compared to non-obese peers [20].

Kurukahvecioglu et al. (2009) reviewed diagnostic challenges in resource-limited settings where access to PSG is restricted by studying a sample of 150 habitual snorers. They discovered that 40% diagnosed clinically with OSA based on symptoms alone did not actually have the condition when tested with PSG later on. The lack of PSG availability resulted in frequent misdiagnoses; particularly in rural areas where incorrect clinical diagnoses reached as high as 60%. PSG identified OSA in 35% of children who did not snore, underscoring its importance for accurate diagnosis. The authors recommended increasing access to PSG or developing alternative diagnostic methods like portable home-based sleep tests to mitigate these errors [21].

The misdiagnosis of pediatric obstructive sleep apnea (OSA) can lead to significant neurobehavioral morbidity, as highlighted by a study conducted by Owens (2009). In this comprehensive review, Owens synthesized data from multiple studies involving over 1,000 participants, finding that 20-30% of children with sleep-disordered breathing (SDB) exhibited neurocognitive deficits, including impaired attention, memory, and executive function. Notably, children with moderate to severe SDB were 3.16 times more likely to have attention deficits (AOR 3.16, 95% CI 1.98-5.02), while those with milder forms had a 15% higher risk of academic underperformance compared to healthy peers. Behavioral issues such as hyperactivity were observed in 40% of children with SDB (AOR 2.82, 95% CI 1.83-4.34), with impulsivity and emotional regulation difficulties occurring at similar rates. The overlap of SDB symptoms with ADHD was emphasized as a significant diagnostic challenge [22].

Bourke et al. (2011) expanded on these findings by examining neurobehavioral outcomes in a cohort of 300 children with varying severities of SDB using standardized neuropsychological tests. The study found that children with mild SDB had 15% lower cognitive performance scores, while those with severe OSA showed a 30% reduction in attention, memory, and executive function abilities. Hyperactivity was present in 50% of children with moderate to severe SDB, while those with primary snoring exhibited a 10% reduction in working memory and attention compared to controls. Additionally, children with moderate to severe SDB were at an increased risk of emotional instability (AOR 2.75, 95% CI 1.84-4.10). The authors stressed that even mild forms of SDB could negatively impact neurocognitive function and advocated for comprehensive evaluations to guide clinical management [23].

Giordani and Chervin (2008) analyzed data from over 2,000 children diagnosed with SDB to examine its neurocognitive and behavioral consequences. They revealed that 30-40% of children with moderate to severe SDB experienced significant attention and learning difficulties, along with an increased risk of behavioral problems such as hyperactivity and aggression (AOR 2.82, 95% CI 1.83-4.34). Additionally, untreated SDB was linked to a 25% higher likelihood of repeating a grade or requiring special education services. The study also found that deficits in executive function - including impaired problem-solving and memory - were most pronounced in cases of severe OSA (Table 1) [24].

**Table 1:** Current Diagnostic Challenges in Pediatric OSA

Author(s)	Sample Size (Type of Patients)	Hyperactivity (% or AOR)	Aggression (% or AOR)	Comorbidities	Challenges
Jin et al. [14]	300 (Pediatric patients aged 4–12 years with snoring)	Not reported	Not reported	Distinct sound patterns in 70% of moderate-severe OSA cases	High false-positive rate (70%) for mild OSA using acoustic analysis alone.
Zancanella et al. [15]	500 (Brazilian children with suspected OSA)	Not reported	Not reported	Nocturnal enuresis (30%), frequent nighttime awakenings (45%)	Clinical symptoms alone insufficient; PSG required for definitive diagnosis.
Certal et al. [16]	2,000+ (Pediatric patients with habitual snoring)	Not reported	Not reported	Higher BMI increased OSA risk 2.5 times	Symptom-based diagnosis had low PPV (60%); clustering symptoms improves accuracy.
Yoon et al. [17]	1,800 (Children with ADHD and suspected sleep disorders)	25% had undiagnosed OSA	Not reported	ADHD; 40% improvement post-OSA treatment	ADHD misdiagnosis delays OSA treatment; PSG vital for accurate diagnosis.
Tsai & Huang [18]	600 (Children aged 5–14 with EDS symptoms)	Not reported	Not reported	EDS linked to poor academic performance (30%)	EDS not reliable in younger children; PSG essential for accuracy.
Owens et al. [19]	400 (Children with suspected OSA)	30% exhibited behavioral issues	Not reported	Genetic predisposition (20%), upper airway resistance (40%)	25% lacked observed apneas; PSG critical for identifying resistant airway cases.
Abrahamyan et al. [20]	5,000+ (Children with suspected OSA)	Not reported	Not reported	Obesity tripled the risk of OSA	Portable monitors had moderate accuracy; models with BMI, neck circumference performed better.
Kurukahvecioglu et al. [21]	150 (Habitual snorers in resource-limited settings)	Not reported	Not reported	OSA identified in 35% of non-snorers via PSG	High clinical misdiagnosis rate (60%); lack of PSG access in rural areas.
Owens [22]	1,000+ (Children with SDB symptoms)	40% exhibited hyperactivity (AOR 2.82)	Emotional regulation difficulties observed	20-30% had neurocognitive deficits; academic underperformance (15%)	SDB symptoms overlap with ADHD; risk of misdiagnosis and neurobehavioral morbidity.
Bourke et al. [23]	300 (Children with varying severities of SDB)	50% with moderate-severe SDB	Not reported	Emotional instability (AOR 2.75); 15% lower cognitive performance in mild SDB	Even mild SDB impacted neurocognitive function; comprehensive evaluations recommended.
Giordani & Chervin [24]	2,000+ (Children with SDB)	30-40% in moderate-severe SDB	Increased risk (AOR 2.82)	25% likelihood of repeating a grade; executive function deficits	Untreated SDB linked to learning difficulties and behavioral issues.

Current diagnosis of pediatric sleep apnea: clinical symptoms

The diagnosis of pediatric obstructive sleep apnea (OSA) begins with a comprehensive understanding of clinical symptoms, which are crucial in identifying children at risk for this condition. One of the most consistently reported symptoms by parents is habitual snoring. Sharara et al. (2010) conducted a clinical observational study involving 290 patients and discovered that persistent snoring under conscious sedation during colonoscopy was significantly associated with OSA, with 65% of those who snored being diagnosed with OSA (p < 0.001). This study emphasizes snoring as an essential predictor of OSA in clinical settings, and such snoring should prompt further diagnostic evaluations, particularly in pediatric populations [25].

Another critical symptom is witnessed apneas during sleep. In a cross-sectional study, McNicholas (2008) evaluated 200 adults suspected of having OSA, using both parental reports of apneas and polysomnography (PSG) as the diagnostic standard. The study found that PSG had a sensitivity of >90% for diagnosing OSA, highlighting its status as the gold standard for OSA diagnosis. Parental reports of apneas, along with symptoms like snoring and excessive daytime sleepiness (EDS), were key clinical indicators [26].

Restless sleep is another frequently observed symptom in children with OSA. A systematic review by DelRosso et al. (2021) involving 1,500 children found that 45% of those with OSA exhibited restless sleep patterns, characterized by frequent movements, gasping, and changes in sleep positions. The review also revealed that this restless sleep was highly correlated with sleep disruption and daytime fatigue (p < 0.01), emphasizing the impact of OSA on sleep architecture [27].

Although excessive daytime sleepiness (EDS) is less common in children than in adults, it is more frequently observed in severe cases of pediatric OSA, particularly in obese children. Gozal and Kheirandish-Gozal (2009) studied 120 children and found that 78% of obese children with OSA exhibited EDS, compared to 45% of non-obese children. The study established a significant link between obesity and EDS in children with OSA (p < 0.01), reinforcing the need for targeted interventions in obese pediatric patients [28].

Behavioral changes such as hyperactivity, irritability, and mood swings are also commonly noted in children with OSA. In a prospective study of 90 children, Mitchell and Kelly (2007) observed that 68% of children who underwent adenotonsillectomy for mild OSA showed significant improvements in behavior, particularly in reducing symptoms that overlap with ADHD, such as hyperactivity and mood swings (p < 0.05). This highlights the therapeutic potential of adenotonsillectomy in addressing both OSA and behavioral disorders in children [29].

Mouth breathing is another significant indicator of OSA, often signaling upper airway obstruction. Li and Lee (2009) conducted a clinical study involving 80 children with suspected sleep-disordered breathing and found that 60% were habitual mouth breathers, which was highly correlated with moderate to severe OSA. The findings stress the importance of evaluating mouth breathing in clinical assessments of pediatric OSA [30].

Nocturnal enuresis (bedwetting) is often linked with pediatric OSA. Brooks and Topol (2003) observed 200 children and found that 41% of those with OSA experienced nocturnal enuresis. After OSA treatment, particularly with adenotonsillectomy or positive airway pressure therapy, the incidence of bedwetting decreased significantly (p < 0.05), highlighting the connection between untreated OSA and enuresis [31].

In addition to physical symptoms, cognitive impairments are also prevalent in children with OSA. Krysta et al. (2017) compared cognitive function in children, adolescents, and adults with OSA, noting that while children exhibited deficits in memory, attention, and executive function, these impairments were less severe than those in adults. The study emphasizes the importance of early diagnosis and intervention to prevent long-term cognitive consequences [32].

Growth delays are another complication arising from untreated OSA. Bonuck et al. (2006) reviewed the literature on growth failure and found that 20% of the 150 children studied exhibited significant growth delays, likely due to disruptions in growth hormone secretion caused by sleep fragmentation. Early treatment of OSA can mitigate these growth issues, underscoring the importance of timely intervention [33].

Parental reports also play a crucial role in diagnosing pediatric OSA, as they often provide detailed observations of sleep disturbances that may not be easily captured in clinical settings. Guilleminault et al. (2004) studied 100 children and found that parental observations of gasping, choking, and witnessed apneas were strongly correlated with polysomnography findings in 75% of cases. This highlights the value of parental input in the diagnostic process [34].

Quality assessment in pediatric OSA

The QUADAS-2 tool is the most commonly used quality assessment tool in the majority of studies evaluating the diagnostic accuracy of pediatric OSA. This tool provides a systematic approach to assess the risk of bias and the applicability of the studies, ensuring a rigorous evaluation of the methodologies used. It evaluates four key domains: patient selection, index test, reference standard, and flow and timing, allowing researchers to identify potential weaknesses in study design.

In addition to QUADAS-2, other diagnostic tools and methods can be utilized for assessing pediatric OSA. The Pediatric Sleep Questionnaire (PSQ) and the OSA-18 Quality of Life Questionnaire are commonly used screening tools that help identify children at risk for sleep-disordered breathing. The PSQ has shown higher sensitivity compared to the OSA-18 questionnaire, making it a valuable tool for initial screening. However, while these questionnaires provide useful insights, they should not replace objective measures such as polysomnography, which remains the gold standard for diagnosing OSA. Other alternative diagnostic methods include home sleep apnea testing (HSAT) and nocturnal pulse oximetry, which can be beneficial in specific populations or settings where polysomnography is not feasible.

The diagnosis of pediatric OSA

The diagnosis of pediatric OSA can be challenging due to the variability in symptoms and the limitations of diagnostic tools. Several studies have evaluated the sensitivity, specificity, and overall effectiveness of different diagnostic methods, including questionnaires and objective measures like polysomnography (PSG) and pulse oximetry (PO).

In the study conducted by Wu et al. (2020), a meta-analysis aimed to compare the pooled sensitivity and specificity of the Pediatric Sleep Questionnaire (PSQ), the OSA-18, and pulse oximetry (PO) in detecting pediatric obstructive sleep apnea syndrome (OSAS) based on severity. This comprehensive analysis included 39 studies with a total of 6,131 children aged 2.9 to 16.7 years, assessing the effectiveness of these tools against the apnea-hypopnea index measured via polysomnography. The results indicated that the PSQ exhibited the highest sensitivity (74%) for detecting mild OSAS. Furthermore, both the PSQ and PO demonstrated comparable sensitivity for moderate and severe OSAS, with PO showing superior specificity in all severity levels (86% for mild, 75% for moderate, and 83% for severe OSAS) [35].

Zong et al. (2022) conducted a study to analyze the correlation between OSA-related quality of life (QoL) and mental health statuses, including depression and anxiety, in adolescent Chinese patients with cleft palate (CP). The researchers utilized the OSA-18, Generalized Anxiety Disorder Scale (GAD-7), and Patient Health Questionnaire-9 (PHQ-9) to assess the impact of OSA on QoL and the mental health of the participants. The study found that 8.7% of the patients reported a moderate to high impact of OSA on their QoL, which was significantly greater than the control group of non-CP adolescents. The mean OSA-18 score was also notably higher in the CP group (36.26 ± 13.50) compared to the control group (28.44 ± 8.93). Additionally, the results indicated that anxiety and depression were more severe in the CP group, with significant positive correlations found between OSA-related QoL and both anxiety and depression statuses [36].

Kainulainen (2020) conducted a doctoral dissertation focused on pulse oximetry-derived biomarkers to assess the severity of obstructive sleep apnea (OSA). The study analyzed both parametric and frequency-domain features of blood oxygen saturation (SpO2) and photoplethysmography (PPG) signals, exploring their associations with daytime sleepiness and impaired vigilance. The findings suggested that pulse oximetry is a valuable tool in the assessment of OSA severity, with potential for improving diagnostic accuracy when combined with clinical evaluations [37].

The meta-analysis by Michelet et al. (2019) investigated the accuracy of the Sleep-Related Breathing Disorder (SRBD) scale in diagnosing obstructive sleep apnea in children. The analysis reviewed various studies to evaluate the sensitivity and specificity of the SRBD scale. The results indicated that while the SRBD scale provides valuable insights, it also has limitations, particularly in terms of specificity [38].

Ferreira-Santos et al. (2022) conducted a systematic review focusing on the application of machine learning techniques for the early diagnosis of obstructive sleep apnea. The study highlighted the potential of machine learning models to analyze large datasets, incorporating clinical features and symptoms to improve diagnostic accuracy. The findings suggest that machine learning approaches may offer a promising alternative to traditional diagnostic tools, potentially enabling earlier and more accurate identification of OSA in pediatric populations [39].

Williams et al. (1991) investigated the effectiveness of combining pulse oximetry with clinical scoring for screening obstructive sleep apnea (OSA). The study found that this combination improved the detection of OSA compared to using either method alone. Pulse oximetry served as an effective non-invasive screening tool, while clinical scores provided valuable context for identifying at-risk individuals [40].

Pediatric obstructive sleep apnea (OSA) is a condition that requires a multifaceted approach to management, tailored to the unique needs of each child. There are various strategies available for managing this condition, ranging from conservative measures to more invasive interventions. These strategies include Conservative Management Strategies, which focus on non-surgical approaches such as lifestyle changes and the use of continuous positive airway pressure (CPAP); Surgical Interventions, which may be necessary for children with anatomical issues contributing to OSA; and Emerging Therapies, which explore new and innovative treatments that aim to improve outcomes for pediatric patients with OSA (Table 2).

**Table 2:** Current Diagnosis Practices of Pediatric OSA

Study	Sensitivity/Specificity	Diagnostic Tools Used	Severity Levels Assessed	OSA-Related QoL	Mental Health Parameters	Innovative Approaches
Wu et al. [35]	Sensitivity: PSQ 74%, PO specificity: 86%	PSQ, OSA-18, Pulse Oximetry	Mild, Moderate, Severe	Not assessed	Not assessed	Polysomnography comparison
Zong et al. [36]	Not reported	OSA-18, GAD-7, PHQ-9	Not assessed	Higher QoL impact in CP group	Anxiety and depression correlations	Not applicable
Kainulainen [37]	Not reported	Pulse Oximetry (SpO2, PPG signals)	Mild, Moderate, Severe	Not assessed	Not assessed	Biomarkers for diagnostic improvement
Michelet et al. [38]	Limited specificity for SRBD	Sleep-Related Breathing Disorder Scale	Not assessed	Not assessed	Not assessed	SRBD scale analysis
Ferreira-Santos [39]	Not reported	Machine Learning Models	Not assessed	Not assessed	Not assessed	Early diagnosis using machine learning
Williams et al. [40]	Improved with combined tools	Pulse Oximetry, Clinical Scoring	Mild, Moderate, Severe	Not assessed	Not assessed	Combined scoring and oximetry screening

Conservative management of pediatric obstructive sleep apnea

Lifestyle modifications play a crucial role in the conservative management of pediatric obstructive sleep apnea (OSA). Weight management is particularly important, especially for overweight or obese children, as excess weight can exacerbate OSA symptoms. A study by Andersen et al. (2019) demonstrated that a structured weight-loss management program significantly improved outcomes in children and adolescents with obesity-related OSA. The study involved 60 participants and reported that those who engaged in the weight-loss program experienced substantial reductions in the apnea-hypopnea index (AHI) and improvements in overall sleep quality [41].

Furthermore, Kang et al. (2012) examined the relationship between body weight status and OSA in children. This study included 200 children and found that obesity was significantly associated with an increased prevalence of OSA. The findings indicated that children with higher body mass indices (BMIs) were at a greater risk for developing OSA, reinforcing the importance of addressing obesity as part of a comprehensive treatment plan [42].

A review by Qian et al. summarized 11 longitudinal studies with 13,550 participants exploring the impact of weight and anthropometric factors on obstructive sleep apnea (OSA). The findings consistently highlight weight gain as a significant risk factor for OSA development and progression. A study in Hong Kong linked a one-unit increase in BMI z-score to higher OSA risk in early adulthood (aOR = 1.54, 95% CI: 1.17-2.02). The Wisconsin Sleep Cohort Study found weight gains of 5%, 10%, and 20% over four years increased the risk of moderate-to-severe OSA (aORs: 2.5, 6.0, and 36.6, respectively). Childhood overweight patterns also elevated OSA risk in middle age, with persistent and incident overweight showing aORs of 1.36 (95% CI: 1.04-1.77) and 1.47 (95% CI: 1.11-1.96), respectively. Weight changes influenced OSA severity, as demonstrated by the Sleep Heart Health Study, which reported significant increases in the respiratory disturbance index (RDI) with weight gains of 5-10 kg and >10 kg. Males experienced a stronger link between weight gain and RDI increases compared to females. Similarly, the Wisconsin Sleep Cohort Study showed that weight gains of 5%, 10%, and 20% increased the apnea-hypopnea index (AHI) by 15%, 32%, and 70%, while weight loss reduced AHI. However, a study in elderly populations found no significant association between BMI or body fat changes and AHI over seven years [43].

Avoidance of allergens and irritants is a critical aspect of managing pediatric obstructive sleep apnea (OSA). Research indicates that exposure to environmental allergens can worsen airway inflammation and contribute to sleep-disordered breathing. Rubinstein and Baldassari (2015) emphasized that managing environmental factors, including allergen exposure, is essential in the comprehensive treatment of pediatric OSA. They noted that children with OSA often have comorbid conditions such as allergic rhinitis, which can exacerbate their symptoms. Effective management strategies should include allergen avoidance to reduce airway inflammation and improve respiratory function [44].

Chirakalwasan and Ruxrungtham (2014) further explored the linkage between allergic rhinitis and OSA, highlighting that children with allergic rhinitis are at an increased risk for developing OSA due to the exacerbation of airway obstruction caused by allergens. Their review suggested that minimizing exposure to common allergens, such as dust mites and pet dander, can significantly benefit children with OSA [45].

A study by Zeng et al., part of the Shanghai Sleep Health Study (SSHS), examined the association between allergic rhinitis (AR)-related genetic polymorphisms and obstructive sleep apnea (OSA) in a cohort of 5322 men with snoring complaints. Comprehensive anthropometric, biochemical, and polysomnographic (PSG) data were analyzed alongside 27 AR-associated single nucleotide polymorphisms (SNPs).

The results revealed that SNP rs12509403 increased the risk for OSA (OR = 1.341, 95% CI = 1.039-1.732, P = 0.024), while rs7717955 reduced the risk (OR = 0.829, 95% CI = 0.715-0.961, P = 0.013). Rs12509403 was significantly associated with higher sleep-breathing parameters, including REM-AHI and NREM-AHI (ORs = 1.496 and 1.471, respectively). Severe OSA cases with rs12509403 demonstrated a greater likelihood of being in higher REM-AHI quartiles [46].

Implementing effective sleep hygiene practices is crucial for managing pediatric obstructive sleep apnea (OSA) and enhancing overall sleep quality. Consistent sleep schedules and a comfortable sleep environment are essential for improving sleep quality and alleviating symptoms associated with OSA.

A study by Lee et al. (2015) investigated the association between sleep hygiene and various factors in patients with mild obstructive sleep apnea, involving 110 patients. The researchers found that poor sleep hygiene was significantly correlated with increased daytime sleepiness and depressive symptoms, with a reported 53% of participants experiencing daytime sleepiness linked to inadequate sleep hygiene practices. The study utilized standardized questionnaires to assess sleep hygiene practices, daytime sleepiness, and depressive symptoms, revealing that participants with good sleep hygiene had a 20% lower score on the Epworth Sleepiness Scale, indicating improved sleep quality and a better quality of life [47].

Similarly, Jung et al. (2019) assessed sleep hygiene-related conditions in patients with mild to moderate OSA, involving 100 participants. The study found that implementing sleep hygiene education significantly improved sleep conditions, with participants who adhered to sleep hygiene practices reporting a 30% reduction in nighttime awakenings and a 25% improvement in overall sleep satisfaction. Notably, participants who maintained a dark, quiet sleep environment and avoided stimulating activities before bedtime experienced a statistically significant reduction in OSA symptoms, with an 18% decrease in the apnea-hypopnea index (AHI) [48].

Furthermore, Bitners and Arens (2020) highlighted the synergistic effect of combining lifestyle modifications with good sleep hygiene practices in managing obstructive sleep apnea syndrome (OSAS) in children. Early recognition and diagnosis of OSAS are crucial due to potential neurobehavioral and cardiovascular consequences. The review emphasizes that effective management should include not only medical interventions like adenotonsillectomy and positive airway pressure (PAP) therapy but also significant lifestyle changes. Weight management is particularly important for overweight or obese children, as weight loss can lead to substantial improvements in OSAS symptoms. Coupled with this, adopting good sleep hygiene practices - such as maintaining a consistent sleep schedule, creating a conducive sleep environment, and avoiding allergens and irritants - can further enhance treatment outcomes. By integrating these lifestyle modifications with proper sleep hygiene, healthcare providers can better address the multifaceted nature of OSAS, ultimately improving the quality of life for affected children [49].

The study by Subramanyam et al. (2020) explored the link between secondhand smoke exposure and the risk of obstructive sleep apnea syndrome (OSAS) in children. Utilizing a cross-sectional design, the researchers assessed a cohort of children through parental questionnaires regarding sleep patterns and secondhand smoke exposure, alongside polysomnography for OSAS diagnosis. The results indicated a significant association, revealing that children exposed to secondhand smoke were more likely to exhibit symptoms of OSAS, such as snoring and daytime sleepiness. The findings suggest that reducing secondhand smoke exposure could serve as a vital preventive measure in mitigating the risk of OSAS among children, highlighting the importance of public health initiatives aimed at decreasing such exposures to improve pediatric sleep health [50].

Medication approaches in the treatment of pediatric obstructive sleep apnea

Nasal Decongestants and Corticosteroids for Nasal Congestion

Nasal congestion is a common issue in children with OSA, and the use of nasal decongestants and corticosteroids can help alleviate symptoms.

McLean et al. (2005) conducted a randomized single-blind placebo- and sham-controlled crossover study to evaluate the efficacy of a topical decongestant and external dilator strip in treating nasal obstruction in 10 patients (nine males; mean age 46 ± 5 years) with obstructive sleep apnea (OSA). All participants had normal acoustic pharyngometry. The study measured the effects of treatment on nasal resistance, mouth breathing during sleep, and OSA severity. Results showed a dramatic and sustained reduction in nasal resistance and a significant decrease in the oral fraction of ventilation during sleep, with an absolute reduction of 30% (95% CI: 12-49). Improvements in sleep architecture were noted during active treatment, along with a modest reduction in OSA severity, indicated by a change in the apnea-hypopnea index (AHI) of 12 (95% CI: 3-22). These findings suggest that addressing nasal obstruction can lead to meaningful improvements in OSA symptoms, highlighting the potential benefits of combining pharmacological and mechanical interventions in this patient population [51].

A randomized, double-blind, placebo-controlled trial by Phoophiboon et al. evaluated the effectiveness of intranasal steroids (fluticasone furoate, 110 mcg/day) in managing moderate to severe obstructive sleep apnea (OSA) with coexisting chronic rhinitis. Conducted at Chulalongkorn University, Thailand, between June 2018 and February 2019, the study enrolled 34 non-obese patients with moderate to severe OSA and chronic rhinitis (total nasal symptom score ≥ 6, BMI < 30 kg/m², modified Mallampati < 3). Participants were randomized to receive either intranasal steroids or a placebo for one month. While the adjusted mean change in apnea-hypopnea index (AHI) did not significantly differ between the two groups (mean difference 11.5 ± 7.9 events/hour; 95% CI: -4.9 to 27.8; p = 0.16), the steroid group demonstrated significant within-group reductions in AHI, non-REM respiratory disturbance index (RDI), total nasal symptom score (TNSS), and the Thai Pittsburgh Sleep Quality Index (PSQI) (p = 0.02, 0.01, 0.003, and <0.001, respectively). Notably, the steroid group experienced a significant reduction in non-supine RDI compared to placebo (56.1 ± 21.9 events/hour; 95% CI: 18.9 to 93.2; p = 0.01) and showed a trend toward reduced wake after sleep onset (WASO) duration (15.3 ± 8.2 minutes; 95% CI: -1.7 to 32.2; p = 0.08). However, there was no significant difference in overall PSQI scores between the groups. These findings suggest that intranasal steroids can effectively reduce respiratory disturbances during non-supine sleep and improve nasal and sleep-related symptoms, making them a potential adjunctive treatment for OSA patients with coexisting nasal inflammation [52].

Additionally, a study by Chan et al. (2020) investigated the use of intranasal mometasone furoate in a cohort of 30 children aged 6 to 18 years with mild OSA. The randomized controlled trial showed significant reductions in nasal obstruction and AHI. Notable improvement in the obstructive apnea-hypopnea index (OAHI) was observed, which decreased from 2.7 ± 0.2 to 1.7 ± 0.3 in the mometasone furoate group compared to a change from 2.5 ± 0.2 to 2.9 ± 0.6 in the placebo group (p = 0.039). Additionally, the oxygen desaturation index (ODI) also improved significantly in the treatment group, changing from -0.6 ± 0.5 to +0.7 ± 0.4 (p = 0.037), suggesting that corticosteroids can effectively manage nasal congestion associated with OSA [53].

A review by Chong et al. (2016) examined the evaluated nine randomized controlled trials (910 participants) to compare different intranasal corticosteroids, doses, and delivery methods for chronic rhinosinusitis with nasal polyps. No significant differences were observed between fluticasone propionate, beclomethasone dipropionate, and mometasone furoate in reducing disease severity or symptoms (very low-quality evidence). High-dose steroids showed only slight improvements in nasal polyp scores but increased the risk of epistaxis compared to low doses (moderate-quality evidence). Evidence from delivery method comparisons was inconclusive due to poor study quality. Overall, no type, dose, or method of corticosteroid proved superior, emphasizing the need for more robust research focusing on long-term outcomes and adverse effects [54].

Antihistamines for Allergies

Antihistamines can also be beneficial in managing pediatric OSA, particularly in children with allergies contributing to nasal congestion.

Goldbart et al. (2012) conducted a double-blind, placebo-controlled trial to assess the efficacy of montelukast, a leukotriene receptor antagonist, in treating pediatric obstructive sleep apnea (OSA). The study involved 46 children diagnosed with OSA via polysomnography. Participants were administered daily oral montelukast (4 or 5 mg) or placebo for 12 weeks, with assessments including polysomnography, parent questionnaires, and adenoid size measurements. Results showed a significant decrease in the obstructive apnea index (OAI), indicating improved respiratory function, along with a reduction in symptoms associated with OSA. Additionally, the montelukast group exhibited a significant decrease in adenoid hypertrophy, suggesting montelukast’s ability to target inflammation linked to airway obstruction in children. It highlights the relevance of addressing allergic inflammation in OSA management, which aligns with later studies, where the anti-inflammatory effects of montelukast were also shown to improve OSA outcomes in children [55].

Mason et al. (2015) conducted a systematic review of the effects of sedatives, hypnotics, and opioids on OSA severity, measured through the apnea-hypopnea index (AHI) and oxygen desaturation index (ODI). Fourteen randomized controlled trials, involving 293 adult participants with mild to moderate OSA, were analyzed. The medications assessed included eszopiclone, zolpidem, remifentanil, and sodium oxybate. The results indicated that eszopiclone (3 mg) and sodium oxybate (4.5 g) significantly reduced AHI compared to placebo, with eszopiclone reducing AHI from 31 ± 5 to 24 ± 4 events per hour. Sodium oxybate reduced AHI by a mean difference of −7.41 events/hour. However, some sedatives, such as zolpidem, were associated with increased central apneas, raising safety concerns. While this study focused on adults, it underscores the importance of considering the safety of pharmacological treatments in OSA, particularly when managing pediatric patients with underlying conditions like allergic rhinitis, as noted in Goldbart et al. (2012). Although focused on adults, this study suggests the need for cautious medication use in OSA, resonating with pediatric studies like Goldbart et al. (2012), which emphasize the importance of considering inflammation and airway obstruction in treatment plans [56].

Kuhle et al. (2020) reviewed the efficacy and safety of anti-inflammatory therapies for pediatric OSA by analyzing five randomized controlled trials involving 240 children aged 1 to 18 years with mild to moderate OSA (AHI 1-30 per hour). The study compared two drug classes: intranasal corticosteroids and oral montelukast. The analysis of three trials (n = 137) comparing intranasal corticosteroids to placebo found uncertainty in AHI reduction (MD −3.18, 95% CI −8.70 to 2.35), with low-certainty evidence. Secondary outcomes such as desaturation index (MD −2.12, 95% CI −4.27 to 0.04) and nadir oxygen saturation (MD 0.59%, 95% CI −1.09 to 2.27) showed moderate-certainty evidence but were not consistently statistically significant. Conversely, two trials (n = 103) evaluating montelukast demonstrated a robust reduction in AHI (MD −3.41, 95% CI −5.36 to −1.45), as well as significant improvements in respiratory arousal index (MD −2.89, 95% CI −4.68 to −1.10) and nadir oxygen saturation (MD 4.07%, 95% CI 2.27 to 5.88). These results were supported by moderate to high-certainty evidence. It reinforces the earlier results by Goldbart et al. (2012), highlighting the importance of targeting inflammatory pathways in pediatric OSA, especially when nasal congestion due to allergies is a contributing factor [57].

Stimulants for Daytime Alertness

In some cases, stimulants may be prescribed to improve daytime alertness in children with OSA, particularly those experiencing excessive daytime sleepiness (EDS) despite treatment.

A review by Chapman et al. (2023) aimed to evaluate the efficacy and safety of modafinil and armodafinil in treating residual sleepiness in patients with obstructive sleep apnea (OSA) who are compliant with continuous positive airway pressure (CPAP) therapy [58]. The European Medicines Agency (EMA) had previously removed modafinil's indication for residual sleepiness in OSA due to concerns over its risk-benefit profile. Researchers reviewed 232 abstracts, ultimately selecting 10 randomized, placebo-controlled trials (1466 patients) for inclusion.

The meta-analysis revealed that modafinil/armodafinil significantly improved daytime sleepiness, with a reduction of 2.2 points on the Epworth Sleepiness Scale (ESS) and a 3-minute increase in the Maintenance of Wakefulness Test (MWT) over placebo. The Multiple Sleep Latency Test (MSLT) and the Functional Outcomes of Sleep Questionnaire (FOSQ) also demonstrated modest improvements. However, modafinil/armodafinil significantly increased adverse events (AEs), including a higher rate of AEs leading to withdrawal (7.2% vs. 3%) and a slight increase in systolic and diastolic blood pressure. Despite these AEs, there was no increase in serious adverse events, such as hospitalizations or death [58].

Another study by Ronnebaum et al. (2021) presented an indirect treatment comparison meta-analysis evaluating the efficacy and safety of solriamfetol, modafinil, and armodafinil for excessive daytime sleepiness (EDS) in obstructive sleep apnea (OSA) patients undergoing continuous positive airway pressure (CPAP) treatment. Six randomized controlled trials (RCTs) involving 1,714 participants were included. The primary efficacy measures were improvements in the Epworth Sleepiness Scale (ESS), the 20-minute Maintenance of Wakefulness Test (MWT20), and the Clinical Global Impression of Change (CGI-C), while the secondary measure assessed improvements in the Functional Outcomes of Sleep Questionnaire (FOSQ). Safety outcomes focused on the incidence of treatment-emergent adverse events (AEs), including serious AEs and those leading to treatment discontinuation. After 12 weeks of treatment, all three medications (solriamfetol, modafinil, and armodafinil) demonstrated greater improvements in ESS, MWT20, and CGI-C compared to placebo. Among the treatments, solriamfetol (150 mg and 300 mg) showed the highest probability of improvement across these efficacy measures. Both modafinil and solriamfetol also significantly improved FOSQ scores relative to placebo. Safety profiles across the treatments were similar, with less than 2% of participants experiencing serious AEs or discontinuation-related AEs, suggesting comparable safety risks [59].

Another study by Kuan et al. presents a systematic review and meta-analysis evaluating the efficacy of modafinil and armodafinil in treating excessive daytime sleepiness (EDS) in patients with obstructive sleep apnea (OSA). Both drugs are wakefulness-promoting agents commonly used in patients who continue to experience residual EDS despite treatment with continuous positive airway pressure (CPAP), the standard first-line therapy for OSA. The researchers reviewed data from randomized controlled trials (RCTs) to assess the impact of these medications on subjective and objective measures of sleepiness. A search of electronic databases, including PubMed, EMBASE, and the Cochrane Central Register of Controlled Trials, identified 11 RCTs of modafinil (involving 723 patients) and five RCTs of armodafinil (involving 1,009 patients). Using a random-effects model, the pooled results demonstrated significant improvements in sleepiness parameters for both medications compared to placebo. Specifically, modafinil reduced Epworth Sleepiness Scale (ESS) scores by a weighted mean difference (WMD) of -2.96, while armodafinil reduced ESS scores by a WMD of -2.63. Additionally, sleep latency as measured by the Maintenance of Wakefulness Test (MWT) was significantly prolonged by both drugs, with a WMD of 2.51 for modafinil and 2.71 for armodafinil [60].

Surgical Interventions: Adenotonsillectomy

Adenotonsillectomy is the first-line surgical intervention for pediatric obstructive sleep apnea (OSA), particularly in children with adenotonsillar hypertrophy. This procedure involves the removal of the tonsils and adenoids, which can significantly increase the size of the upper airway and reduce the likelihood of airway collapse during sleep.

The study conducted by Li et al. focused on assessing the efficacy and safety of adenotonsillectomy for treating obstructive sleep apnea (OSA) in children with Down syndrome (DS). Given that OSA is prevalent in children with DS and impacts their physical and psychological growth, the researchers aimed to explore how effective adenotonsillectomy is in this specific group, as it is the primary treatment for pediatric OSA. The study involved a systematic review and meta-analysis of nine studies, encompassing a total of 384 participants. The main outcomes analyzed were changes in the apnea-hypopnea index (AHI), minimum oxygen saturation, sleep efficiency, and arousal index through polysomnography. Results showed a significant decrease in AHI by 7.18 events per hour (95% CI: -9.69, -4.67; p < 0.00001), reflecting a reduction in OSA severity. The minimum oxygen saturation improved by 3.14% (95% CI: 1.44, 4.84; p = 0.0003), indicating better oxygen levels during sleep. However, sleep efficiency showed no significant improvement (MD 1.69%; 95% CI: -0.59, 3.98%; p = 0.15), while the arousal index decreased by 3.21 events per hour (95% CI: -6.04, -0.38; p < 0.03), suggesting fewer interruptions during sleep. The success rate for achieving a postoperative AHI of less than 1 was 16% (95% CI: 12%-21%), and for an AHI of less than 5, the success rate was 57% (95% CI: 51%-63%). Despite the benefits, residual OSA and complications, such as airway obstruction and bleeding, were noted, which calls for further exploration [61].

The similar study by Lee et al. focused on evaluating the effectiveness of adenotonsillectomy for treating obstructive sleep apnea (OSA) in children with Prader-Willi syndrome (PWS). The researchers conducted a meta-analysis of six studies involving 41 children (mean age: 5 years, 55% boys) to clarify the surgical outcomes. The findings showed a significant decrease in the apnea-hypopnea index (AHI) from 13.1 events per hour (95% CI: 11.0-15.1) before surgery to 4.6 events per hour (95% CI: 4.1-5.1) postoperatively. The net reduction in AHI was 8.0 events per hour (95% CI: -10.8 to -5.1). The overall success rate for achieving a postoperative AHI of less than 1 was 21% (95% CI: 11%-38%), and for a postoperative AHI of less than 5, it was 71% (95% CI: 54%-83%) [62].

Despite the improvements, residual OSA was frequently observed, and some patients developed velopharyngeal insufficiency after surgery. The study concluded that adenotonsillectomy is effective in reducing OSA severity in children with PWS, but residual OSA remains a concern that requires ongoing attention.

The study by Clements et al. is a systematic review and meta-analysis that evaluates the outcomes of adenotonsillectomy in treating obstructive sleep apnea (OSA) among children with Prader-Willi syndrome (PWS), a condition that predisposes them to OSA due to obesity, hypotonia, and abnormal ventilatory responses. The analysis included 68 patients from eight studies, with 46 of those patients having reported individual preoperative and postoperative polysomnography (PSG) results. The mean postoperative improvement in the apnea-hypopnea index (AHI) was significant, with a reduction of 7.7 events per hour (95% CI: 4.9-10.5). Postoperatively, 20% of patients had an AHI of less than 1.5, indicating complete resolution of OSA, while 67% showed improvement from moderate or severe OSA to mild or resolved cases (AHI < 5). However, the study also found that 24% of the patients experienced postoperative complications, with velopharyngeal insufficiency being the most common (14%). Despite improvements in AHI and quality of life, many patients had residual OSA post-surgery [63].

In the study by Tanna and Choi (2009), a retrospective analysis was conducted to evaluate the efficacy and safety of adenotonsillectomy for pediatric obstructive sleep apnea (OSA) in children with Prader-Willi syndrome (PWS). The study took place in a tertiary care children's hospital and included three PWS patients (two female twins and one boy), all of whom underwent adenotonsillectomy between January 2004 and December 2005. Preoperative and postoperative full overnight polysomnography (PSG) reports were analyzed, and postoperative complications were recorded. The results were variable. While one patient with the highest body mass index and largest tonsils showed significant improvement in OSA, the resolution was inconsistent across all patients. The postoperative apnea-hypopnea index (AHI) results were 13.3, 2.4, and 1.6 events per hour, respectively, indicating that some residual OSA remained post-surgery. Despite this, no perioperative complications or adverse events were reported, and all patients were discharged within 24 hours postoperatively [64].

Lingual Tonsillectomy

Lingual tonsillectomy is a surgical option for treating pediatric obstructive sleep apnea (OSA), particularly in cases of persistent OSA after adenotonsillectomy caused by lingual tonsil hypertrophy.

The retrospective case series study by Skirko et al. (2019) evaluated the perioperative course and morbidity in children with Down syndrome (DS) who underwent lingual tonsillectomy (LT) for residual obstructive sleep apnea (rOSA) from April 2011 to July 2016. The study included 39 patients, with a mean hospital stay of 1.3 days and a low complication rate. Only one patient (2.6%) experienced a postoperative bleed, which did not require surgical intervention. Minor complications, such as periodic oxygen desaturation, were observed in 41% of patients, but no major complications occurred. The effectiveness of LT in improving rOSA was mixed. Among 29 patients with available postoperative data, the median obstructive apnea-hypopnea index (OAHI) decreased from 11 to 7 (p = 0.07), with 32% of subjects achieving an OAHI < 5, and only 17% being cured of OSA (OAHI < 2). The lowest oxygen saturation improved from 78% to 82% postoperatively (p = 0.003). The study concluded that while LT has a favorable postoperative course, its effectiveness in curing rOSA in DS patients remains limited, suggesting further research is necessary to optimize surgical management [65].

The study by Prosser et al. (2017) evaluated the outcomes of lingual tonsillectomy (LT) in children with Down syndrome (DS) who had persistent obstructive sleep apnea (OSA) following adenotonsillectomy (T&A). A retrospective chart review was conducted on 40 DS patients who underwent LT between 2003 and 2013, with 21 meeting the inclusion criteria of having both pre- and post-operative polysomnography (PSG). The median apnea-hypopnea index (AHI) decreased significantly from 9.1 to 3.7 events/hour (P < .0001), while the median obstructive AHI (oAHI) improved by 5.3 events/hour. Additionally, the mean oxygen saturation nadir improved from 84% to 89% (P = .004), though there were no significant changes in CO2 levels or central apnea index. Post-operatively, 61.9% of patients had an oAHI of less than 5 events/hour, while 19% achieved an oAHI below 1 event/hour. The study concluded that LT significantly improves AHI, oAHI, and oxygen saturation nadir in children with DS who have persistent OSA following T&A [66].

The study by DeMarcantonio et al. reviewed the safety and efficacy of lingual tonsillectomy (LT) in children with persistent obstructive sleep apnea (OSA) after adenotonsillectomy. Analyzing data from 92 children (mean age 8.6 years, 50% female), the results indicated a significant reduction in the apnea-hypopnea index (AHI), which decreased from a median of 8.5 to 3.8 events per hour (p = 0.022). The obstructive AHI (oAHI) also showed a notable reduction from 8.3 to 3.1 events per hour (p = 0.021). Moreover, the oxygen saturation nadir improved from 83.8% to 89.0% (p = 0.0007). Postoperatively, 61.1% of patients achieved an oAHI of less than 5 events per hour, compared to 27.8% preoperatively (p = 0.032). The complication rates included a 4.4% readmission rate and a similar rate of emergency department visits for bleeding (4.4%) and poor oral intake (3.3%). Overall, the study concluded that LT is effective in significantly reducing OSA severity in children, with complication rates comparable to traditional tonsillectomy, indicating its potential as a valuable surgical option for this patient population [67].

The systematic review and meta-analysis by Rivero and Durr examined the role of lingual tonsillectomy (LT) in pediatric patients experiencing persistent obstructive sleep apnea (OSA) following tonsillectomy and adenoidectomy (T&A). The analysis included five retrospective case series with a total of 132 patients, focusing on key outcome measures such as the apnea-hypopnea index (AHI) and minimum oxygen saturation (minSaO2). Results demonstrated a statistically significant mean reduction in AHI of -6.64 (95% CI, -8.63 to -4.65) and a mean increase in minSaO2 of 4.17 (95% CI, 1.25-7.08), highlighting the efficacy of LT in improving respiratory outcomes [68].

However, the overall success rate, defined as achieving a postoperative AHI of less than 5, was only 52%, indicating that while LT can be an effective adjunctive procedure, complete resolution of OSA is uncommon. The study’s findings align with other research on LT, suggesting a consistent trend toward reduced AHI and improved oxygen saturation across various patient populations undergoing lingual tonsillectomy.

Lingual tonsil hypertrophy can be assessed using computed tomography, magnetic resonance imaging, sleep endoscopy, or cine magnetic resonance imaging. Lingual tonsillectomy is typically performed under endoscopic visualization using techniques such as radiofrequency ablation (Coblation), microdebrider, or carbon dioxide laser.

Palatal Surgery

Palatal surgery for the treatment of obstructive sleep apnea (OSA) has undergone significant advancements, with modern techniques shifting toward less invasive approaches that improve patient outcomes. The primary goal of these interventions is to widen the soft palate, thus improving airflow and reducing airway obstruction during sleep. Common procedures include uvulopalatopharyngoplasty (UPPP), expansion sphincter pharyngoplasty, and palatal implants - each targeting distinct areas of the palate to alleviate OSA symptoms (Yaremchuk, 2016). These surgeries have demonstrated effectiveness in adults, but their application in pediatric populations is less frequent due to concerns about their potential effects on growth and development. However, innovations in tissue-preserving techniques, such as palatal hybrid surgery, which integrates multiple surgical strategies for precise airway reconstruction, offer promising results for both adults and select pediatric cases (Li et al., 2022).

Li et al. (2022) investigated the safety and efficacy of palatal hybrid surgery, a tissue-specific approach designed to address velopharyngeal obstruction in OSA patients. The study, which included 46 patients, employed techniques such as mucosa preservation, tonsil excision, fat ablation, and muscle relocation/suspension to reconstruct the tonsillar fossa and advance the uvula. Results demonstrated a substantial reduction in the apnea-hypopnea index (AHI) from 41.8 to 18.2 events/h and an increase in minimum oxygen saturation from 72.4% to 81.5% (p < 0.001). Patient outcomes also showed significant improvements in snoring severity and daytime sleepiness, with an overall success rate of 63%. These findings reinforce the effectiveness of minimally invasive approaches in palatal surgery, especially in early-stage OSA patients [69].

In a broader review of palatal surgery, Li (2019) emphasized the shift from radical excision techniques to functional reconstruction in OSA treatment. The study underscored the core value of palatal surgery in reconstructing the airway, restoring airflow, and rehabilitating muscles, noting that modern techniques, such as relocation pharyngoplasty and suspension palatoplasty, prioritize functional outcomes while minimizing patient morbidity. This shift aligns with the results from Li et al. (2022), as both studies highlight the importance of tissue preservation and the less invasive nature of current surgical approaches [70].

The transformation of palatal surgery is further supported by the work of Yaremchuk (2016), who reviewed the historical development of uvulopalatopharyngoplasty (UPPP), the first widely used surgical intervention for OSA, introduced in 1981. UPPP was initially effective in patients with retro-palatal obstruction but was associated with high morbidity and variable success rates, prompting the development of newer, less invasive techniques. These techniques, including cautery-assisted palatal stiffening operations, laser-assisted uvulopalatoplasty, and radiofrequency volumetric tissue reduction, aim to improve patient outcomes by targeting specific tissues while reducing the invasiveness of the surgery, which is consistent with the findings from Li’s 2019 review on functional reconstruction [71].

Adding to the discussion on patient-specific outcomes, Gul et al. (2024) conducted a prospective cohort study on the impact of palatal surgeries on eustachian tube function in 96 OSA patients. This study examined the effects of three types of palatal surgeries - anterior palatoplasty (AP), expansion sphincter palatoplasty (ESP), and barbed palatoplasty (BP) - on eustachian tube dysfunction symptoms. The results revealed a significant improvement in eustachian tube function over time (p < 0.001), though differences between the surgical groups were not statistically significant. However, patients in the barbed palatoplasty group exhibited higher dysfunction scores at the three-month follow-up, highlighting the need for individualized surgical planning based on OSA severity and anatomical considerations. This study complements the findings of Li et al. (2022) and Li (2019), which emphasize the importance of customized surgical interventions to optimize treatment outcomes in OSA patients [72].

Upper Airway Stenting for Pediatric Obstructive Sleep Apnea

The exploration of upper airway stenting as a non-invasive mechanical alternative for treating OSA in patients who are non-compliant with continuous positive airway pressure (CPAP) therapy has gained attention in recent years. Three notable studies provide a comprehensive evaluation of the efficacy and application of nasopharyngeal stents in managing OSA, each contributing unique insights into this treatment method.

Traxdorf et al. (2016) conducted a single-center case series investigating the first-night efficacy of a nasopharyngeal stent compared to standard nCPAP-titration in eight participants with untreated OSA (prestudy AHI ≥10). Their results demonstrated that the mean AHI was significantly reduced from 31.1 ± 12.0 to 19 ± 12.0 with the stent, although nCPAP showed a more substantial reduction to 8.2 ± 11.9. Despite this, the stent reduced the number of obstructive apneas by over 94%, comparable to surgical interventions like uvulopalatopharyngoplasty, with a 50% responder rate. The absence of complications and high tolerability highlighted the stent as a promising option for patients non-compliant with CPAP, suggesting a widened therapeutic range [73].

In a parallel vein, Woodson et al. (2014) evaluated the efficacy of hypoglossal nerve stimulation, a neuromodulatory approach to OSA management. Their randomized controlled therapy withdrawal study on 46 participants, following 12 months of upper airway stimulation, demonstrated significant improvements in AHI, oxygen desaturation index, and sleep quality measures at 12 and 18 months. Notably, the study indicated that withdrawing therapy led to a return to baseline levels of OSA severity, whereas maintenance of the therapy sustained improvements. While the mechanism differs from stenting, this study underscores the potential of targeting specific anatomical components in OSA therapy, similar to the targeted approach in nasopharyngeal stenting [74].

Dellweg et al. (2022) extended the research on stenting by evaluating the Naśtent's effectiveness in reducing palatal obstructions in OSA patients. Conducted on 101 patients who could not tolerate CPAP, the study demonstrated a significant reduction in the apnea-hypopnea index for patients with anteroposterior palatal collapse, aligning with the findings of Traxdorf et al. regarding the effectiveness of stents in patients with targeted obstruction patterns. However, the Naśtent was not effective in addressing retrolingual or multilevel obstructions, indicating the importance of patient selection in stent-based therapy. Additionally, this study introduced the diagnostic utility of stents, using the Naśtent as a screening tool when drug-induced sleep endoscopy (DISE) was unavailable, with an 85.7% detection rate for soft palate obstructions [75].

Emerging therapies for pediatric obstructive sleep apnea

Hypoglossal Nerve Stimulation

In recent years, Hypoglossal Nerve Stimulation (HGNS) has emerged as a minimally invasive therapeutic intervention for Obstructive Sleep Apnea (OSA). This procedure entails the implantation of a pulse generator that delivers targeted electrical stimulation to the hypoglossal nerve, promoting upper airway muscle activity during sleep to prevent airway collapse. Clinical studies have demonstrated that HGNS effectively reduces the apnea-hypopnea index (AHI) and enhances overall respiratory function. Additionally, patient-reported outcomes indicate significant improvements in sleep quality, daytime alertness, and quality of life metrics.

Hypoglossal Nerve Stimulation (HGNS) has emerged as a promising treatment modality for Obstructive Sleep Apnea (OSA). The foundational work by Schwartz et al. (2001) provided critical early evidence for the efficacy of HGNS. In their study, eight patients with OSA underwent HGNS, resulting in an average reduction in the Apnea-Hypopnea Index (AHI) by 42%, from a baseline AHI of 34.5 to 20.1. Additionally, they reported improvements in oxygen saturation, demonstrating that HGNS effectively reduces airway collapse during sleep and contributes to enhanced sleep quality [76].

Building on these initial findings, Kezirian et al. (2010) conducted a comprehensive review of the literature, affirming that HGNS consistently reduces AHI across multiple studies. This review highlighted the significant variability in outcomes based on patient selection, noting that the success of HGNS is particularly notable in individuals with lower body mass indices (BMIs) and specific anatomical features. The evidence suggested that HGNS could provide an alternative for patients intolerant to Continuous Positive Airway Pressure (CPAP) therapy, thus broadening treatment options for OSA management [77].

Friedman et al. (2016) further advanced the clinical understanding of HGNS by presenting the results of a multicenter study involving 126 patients. Their findings indicated a remarkable 68% reduction in AHI at the six-month follow-up, with AHI decreasing from a baseline of 35.4 to 11.4. This study also assessed quality of life measures, revealing significant improvements in the Epworth Sleepiness Scale (ESS) scores, which dropped from an average of 11.9 to 5.0. Furthermore, 82% of participants reported satisfaction with their treatment, indicating high tolerability and effectiveness of HGNS as a long-term solution for OSA [78].

Most recently, Kent et al. (2019) provided a large-scale evaluation involving 300 patients to assess HGNS treatment efficacy. Their results corroborated earlier findings, with an average AHI reduction of 65%, from 33.5 at baseline to 11.6 after treatment. Importantly, this study identified key predictors of success, emphasizing the significance of anatomical evaluation. Patients with non-collapsing retrolingual airway sites had markedly better outcomes, reinforcing the necessity for thorough preoperative assessment [79].

The consistent findings across diverse patient populations highlight the importance of patient selection, suggesting that HGNS is particularly beneficial for patients who are not ideal candidates for CPAP or surgical interventions.

Oral Appliances

Oral appliances, designed to advance the lower jaw and maintain airway patency during sleep, have shown promising results in treating pediatric OSA. In a systematic review and meta-analysis by Marciuc et al. (2023), which included 12 studies with a total of 580 pediatric patients aged 6 to 18 years, the use of dental appliances resulted in a significant reduction in the apnea-hypopnea index (AHI) by an average of 13.42 events per hour (95% CI: 9.98 to 16.86), leading to normalization of AHI in many children. The study also assessed outcomes like snoring severity and daytime sleepiness, showing improvements in both parameters. Similarly, Ma et al. (2023) reported that mandibular advancement appliances reduced AHI by an average of 7.29 events per hour (p < 0.001) in a cohort of 120 children aged 7 to 16 years, along with improvements in oxygen saturation levels (mean increase of 4.5% during sleep), indicating enhanced respiratory function. Furthermore, Yu et al. (2023) evaluated orthodontic appliances in a network meta-analysis that included 15 trials with 750 pediatric subjects, finding a substantial reduction in AHI by up to 60% (p < 0.001) and significant improvements in symptoms related to OSA, such as snoring and daytime sleepiness, measured by the Epworth Sleepiness Scale. Collectively, these studies highlight the effectiveness of oral appliances in reducing AHI and improving clinical outcomes, supporting their use as a non-invasive alternative for pediatric patients who may not tolerate CPAP therapy [80-82].

Gene Therapy

Gene therapy has emerged as a promising avenue for addressing pediatric obstructive sleep apnea (OSA), with recent research focusing on epigenetic alterations and their potential therapeutic implications. One such study by Cheung et al. (2021) investigated the role of epigenetic alterations in pediatric sleep apnea. The researchers examined DNA methylation, histone modifications, and noncoding RNAs as key mechanisms that may contribute to the development of OSA. Using genome-wide methylation analysis on pediatric patients with OSA, they identified specific epigenetic changes associated with the severity of the disease. Their findings suggest that these epigenetic markers could serve as targets for gene therapy, offering a new, non-invasive treatment approach.

In another study by Perikleous et al. (2018), the authors provided an overview of the role of DNA methylation in pediatric OSA, highlighting preliminary findings from various studies. Their work underscores the importance of epigenetic changes, particularly DNA methylation, in the pathogenesis of OSA. They reviewed data showing that hypermethylation in specific genes may contribute to inflammation and immune responses, which are linked to airway obstruction and OSA severity. The study also suggests that these methylation patterns could be used as biomarkers for early detection and treatment monitoring. This could pave the way for the development of personalized gene therapies that target specific methylation sites, offering a tailored approach to treating pediatric OSA (Table 3) [83-84].

**Table 3:** Treatment approaches for OSA

Treatment Approach	Mechanism of Action	Indications	Considerations
Medical Management			
Nasal Decongestants	Vasoconstriction, reducing nasal congestion	Mild OSA with significant nasal congestion, Seasonal allergic rhinitis	Short-term use, potential rebound congestion
Corticosteroids	Anti-inflammatory, reducing nasal mucosal edema	Chronic rhinitis affecting airflow, Persistent nasal obstruction	Long-term use may require monitoring
Antihistamines	Block histamine receptors, reducing allergic symptoms	Allergies contributing to nasal congestion, Coexisting allergic rhinitis	Sedation as a potential side effect
Stimulants	Increase alertness and attention	Excessive daytime sleepiness (EDS) due to OSA, Attention deficits	Monitor for potential side effects, such as insomnia and decreased appetite
Surgical Interventions			
Adenotonsillectomy	Removal of enlarged tonsils and adenoids	Moderate to severe OSA with adenotonsillar hypertrophy, Failed medical management	May require additional surgery if OSA persists
Lingual Tonsillectomy	Removal of enlarged lingual tonsils	Persistent OSA after adenotonsillectomy, Obstruction due to lingual tonsil hypertrophy	Less common procedure, requires specialized expertise
Palatal Surgery	Restructuring the soft palate to improve airway patency	OSA not resolved by adenotonsillectomy, Structural abnormalities	May have speech or swallowing issues as potential side effects
Emerging Therapies			
Hypoglossal Nerve Stimulation	Stimulates hypoglossal nerve to improve airway patency	Severe OSA in non-surgical candidates, Failed CPAP therapy	Requires surgical implantation and device maintenance
Oral Appliances	Advances mandible and tongue to open airway	Mild to moderate OSA, Intolerance to CPAP	May cause discomfort or dental issues
Gene Therapy	Targets genetic factors contributing to airway obstruction	Experimental, future potential	Still in early stages of research and development

## Conclusions

The management of pediatric obstructive sleep apnea (OSA) has advanced significantly, offering a range of strategies to meet diverse patient needs. Conservative measures, such as lifestyle changes and oral appliances, provide relief for many, while adenotonsillectomy remains the first-line treatment for children with adenotonsillar hypertrophy, demonstrating high success rates. Emerging therapies like hypoglossal nerve stimulation and gene therapy show promise for complex cases or those unresponsive to traditional treatments. Treatment outcomes are influenced by factors such as age, OSA severity, obesity, and craniofacial abnormalities, highlighting the need for personalized approaches. Despite progress, existing research faces limitations, including small sample sizes, varied methodologies, and short follow-up periods. Future studies should address these gaps through larger multicenter trials, improved diagnostic tools, and innovative treatments, such as machine learning for early diagnosis. Early diagnosis is crucial to prevent severe consequences like neurobehavioral and cardiovascular issues. Tailored, multidisciplinary care and ongoing follow-up are essential to optimize outcomes and address recurrence, ensuring effective management of pediatric OSA.
